# Sustained Intratumoral Administration of Agonist CD40 Antibody Overcomes Immunosuppressive Tumor Microenvironment in Pancreatic Cancer

**DOI:** 10.1002/advs.202206873

**Published:** 2023-01-19

**Authors:** Hsuan‐Chen Liu, Daniel Davila Gonzalez, Dixita Ishani Viswanath, Robin Shae Vander Pol, Shani Zakiya Saunders, Nicola Di Trani, Yitian Xu, Junjun Zheng, Shu‐Hsia Chen, Corrine Ying Xuan Chua, Alessandro Grattoni

**Affiliations:** ^1^ Department of Nanomedicine Houston Methodist Research Institute 6670 Bertner Ave Houston TX 77003 USA; ^2^ Texas A&M University College of Medicine 2121 W Holcombe Blvd Houston TX 77003 USA; ^3^ Center for Immunotherapy Research Houston Methodist Research Institute 6670 Bertner Ave Houston TX 77003 USA; ^4^ ImmunoMonitoring Core Houston Methodist Research Institute 6670 Bertner Ave Houston TX 77003 USA; ^5^ Department of Surgery Houston Methodist Hospital 6565 Fannin St. Houston TX 77003 USA; ^6^ Department of Radiation Oncology Houston Methodist Hospital 6565 Fannin St. Houston TX 77003 USA

**Keywords:** drug delivery, immunotherapy, implantable device, pancreatic cancer, sustained release

## Abstract

Agonist CD40 monoclonal antibodies (mAb) is a promising immunotherapeutic agent for cold‐to‐hot tumor immune microenvironment (TIME) conversion. Pancreatic ductal adenocarcinoma (PDAC) is an aggressive and lethal cancer known as an immune desert, and therefore urgently needs more effective treatment. Conventional systemic treatment fails to effectively penetrate the characteristic dense tumor stroma. Here, it is shown that sustained low‐dose intratumoral delivery of CD40 mAb via the nanofluidic drug‐eluting seed (NDES) can modulate the TIME to reduce tumor burden in murine models. NDES achieves tumor reduction at a fourfold lower dosage than systemic treatment while avoiding treatment‐related adverse events. Further, abscopal responses are shown where intratumoral treatment yields growth inhibition in distant untreated tumors. Overall, the NDES is presented as a viable approach to penetrate the PDAC immune barrier in a minimally invasive and effective manner, for the overarching goal of transforming treatment.

## Graphical abstract

Nanofluidic drug‐eluting seed (NDES) enables long‐term intratumoral immunotherapeutic treatment. Sustained low‐dose agonist CD40 monoclonal antibody delivered via NDES effectively triggers anti‐tumor immune response in immunosuppressive pancreatic tumor microenvironment without treatment‐related adverse events. Overall, the NDES presents an effective low‐dose tumor‐targeted drug delivery approach for enhanced treatment efficacy and survival.

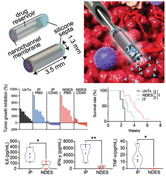

## 1 Introduction

Pancreatic ductal adenocarcinoma (PDAC) is an aggressive cancer of the exocrine pancreas, which is frequently diagnosed at advanced stages. With a 5‐year survival rate of less than 7% and median survival of 4–6 months,^[^ ^1^ ^]^ there is a dire need for improved early detection and treatment. About 85% of patients present with advanced or metastatic disease^[^ ^2^ ^]^ and less than 20% of patients are eligible for surgical resection. Adjuvant chemotherapy with gemcitabine or combination regimens (i.e., FOLFIRINOX: irinotecan, oxaliplatin, 5‐fluorouracil, and leucovorin) only moderately prolong life expectancy.^[^ ^3^ ^]^ The aggressive nature and intrinsic chemotherapy resistance of PDAC limit treatment success,^[^ ^4^ ^]^ thus necessitating improved therapeutic strategies.

PDAC inherently have an immunosuppressive tumor immune microenvironment (TIME).^[^ ^5^ ^]^ The TIME of PDAC is infiltrated with T‐regulatory cells (Tregs), M2 polarized tumor‐associated macrophages (TAMs), and myeloid‐derived suppressive cells, which block anti‐tumor activities of effector CD4+ and CD8+ T cells.^[^ ^6^ ^]^ Immunotherapy, inclusive of immune checkpoint inhibitors (ICIs) and agonist monoclonal antibodies (mAb), is a promising treatment avenue, as it harnesses the host immune system to target the TIME. Immunotherapy generates anti‐tumor immunogenic responses through the interaction between antigen presenting cells (APC) and T cells. APCs induce T cell priming, where activated T cells recognize tumor‐specific antigens and incite tumor‐targeted cytotoxicity.^[^ ^7^ ^]^ In clinical trials, single agent ICIs targeting CTLA‐4 or PD‐1/PD‐L1 did not improve outcomes in unselected patients with PDAC.^[^ ^8^ ^]^ In contrast, agonistic agents acting as host immune response initiator, such as CD40, ICOS, and 4‐1BB, could serve as a potent immunotherapy avenue for PDAC therapy. Further, recent studies highlighted the synergistic effects of nanomedicine approaches and immunotherapy as potential avenues for improving therapeutic outcomes of PDAC.^[^ ^9^ ^]^

Of note, CD40 activation of APCs is critical for T cell licensing and promoting antigen‐specific T cell responses.^[^ ^10^ ^]^ CD40 is a tumor necrosis factor (TNF) receptor member expressed on many immune cell types, including dendritic cells (DCs), macrophages, B cells, and natural killer (NK) cells.^[^ ^11^ ^]^ In line with this, CD40 agonist mAb can initiate cytotoxic immunity in the TIME of PDAC. Current clinical trials are exploring the therapeutic impact of CD40 agonist mAb including selicrelumab, sotigalimab, and dacetuzumab.^[^ ^12^ ^]^ In a phase I clinical study, neoadjuvant selicrelumab treated resectable PDAC tumors was compared to untreated or chemotherapy/chemoradiation‐treated tumors (NCT02588443).^[^ ^13^ ^]^ Selicrelumab‐treated tumors showed T cell enrichment and fibrosis reduction with fewer M2‐like macrophages and more mature DCs. A phase 1b clinical trial evaluated the safety of sotigalimab in combination with standard chemotherapy, gemcitabine plus nab‐paclitaxel, with or without nivolumab, in patients with metastatic PDAC (NCT03214250). The result indicated that this combinatorial regimen can potentially replace chemotherapy‐only standard in metastatic PDAC; however, most participants had grade 3 or 4 treatment‐related adverse events (TRAE).^[^ ^14^ ^]^ Specifically, cytokine storm, hepatotoxicity, and thromboembolic events were reported, which result in dose‐limiting treatment efficacy.^[^ ^15^ ^]^ While some adverse events are clinically controllable, improving therapeutic index could vastly enhance clinical outcome.

To circumvent systemic drug exposure and its related toxicities, local drug delivery is increasingly investigated.^[^ ^16^ ^]^ Local delivery is predicated on the premise that confining drugs to the TIME increases bioavailability, thus improving efficacy while minimizing systemic toxicities.^[^ ^16a–c^ ^]^ In preclinical studies, intratumoral delivery of CD40 agonist mAb demonstrated treatment efficacy with reduced toxicity in murine bladder cancer,^[^ ^17^ ^]^ melanoma,^[^ ^18^ ^]^ and colon cancer.^[^ ^19^ ^]^ Clinically, several trials of local CD40 agonist mAb delivery are under investigation for various cancers (NCT02706353, NCT NCT04059588, NCT05126472, NCT04547777).

Effective local delivery necessitates precise injection into the tumor and hinges on confining drugs locally for an extended duration. However, as it stands, current clinical variations in intratumoral administration approaches include different injection techniques (needle type, needle gauge size, injection volume, and speed), which could affect delivery and consequently, treatment efficacy.^[^ ^20^ ^]^ Regardless of technique, bolus volume injection rapidly diffuses out of the tumor, mimicking systemic administration, where cytokine storm can occur within several minutes.^[^ ^20^, ^21^ ^]^ Further, microenvironment factors such as tumor composition, vascularity, and interstitial fluid pressure can markedly affect intratumoral drug distribution and retention. Currently in the clinic, repeated intratumoral injections are necessary in part attributable to rapid tumor leakage, which could be challenging for hard‐to‐reach tumors or patients in a fragile state of health.^[^ ^20b^ ^]^ Depending on the lesion type and location, each injection could entail sedation, anesthetic and intubation.^[^ ^22^ ^]^ On this note, some PDAC patients received up to 80 intratumoral injections over the course of ≈2 months under endoscopic ultrasound guidance.^[^ ^22^ ^]^ Needless to say, repeated procedures increase healthcare burden, costs, and risks of infections. As such, these cumulative challenges could severely limit widespread clinical adoption.

To this end, we surmise that a single‐administration extended‐released platform for intratumoral drug delivery, one that avoids repeated injections or variations in administration technique, could be a clinically viable approach. In this study, we delivered CD40 agonist mAb intratumorally via a local controlled release nanofluidic drug‐eluting seed (NDES)^[^ ^23^ ^]^ in murine PDAC models. As opposed to large volume bolus injection, the NDES releases drug molecules via concentration‐driven diffusion in a slow and controlled manner over a timeframe of weeks. Further, the NDES is intratumorally implanted in a one‐time minimally invasive manner using a clinical trocar approach akin to brachytherapy insertion. Here, we hypothesize that sustained intratumoral drug delivery will reduce adverse reactions associated with systemic CD40 mAb exposure and local administration will enhance bioavailability to improve immune activation against PDAC.

## 2 Results

### 2.1 Sustained Local Administration of Agonist CD40 Monoclonal Antibodies Demonstrated Effective Tumor Reduction

**Figure 1:**
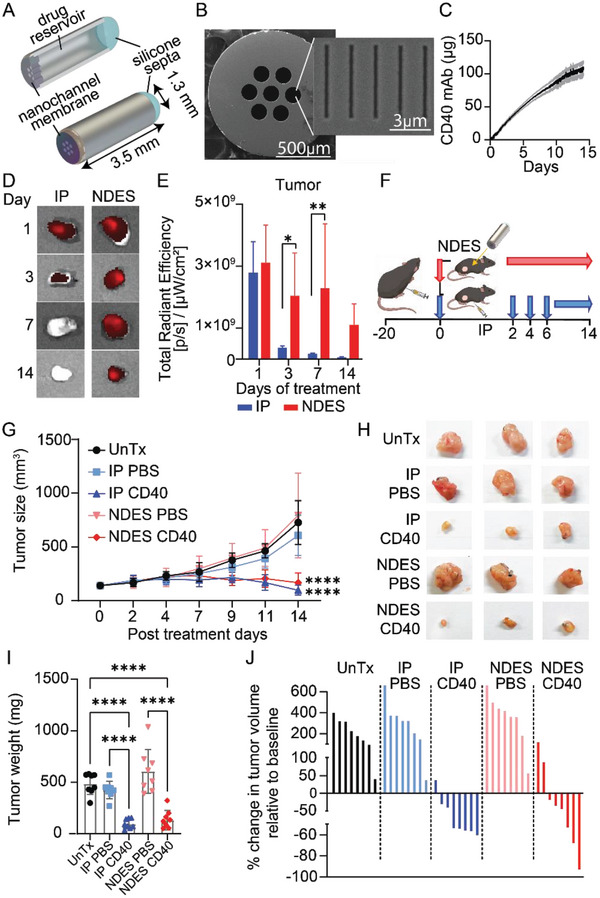
KPC tumor‐bearing mice were treated with CD40 mAb via systemic (IP) or local (NDES) administration. A) Rendering of NDES components. B) Scanning electron microscopy (SEM) image of nanochannels in the nanofluidic membrane. C) In vitro cumulative release of CD40 mAb from NDES (*n* = 4). Data are expressed as mean ± standard deviation. D) Representative ex vivo tumor IVIS images (*n* = 6). E) Bar‐graph depicted the CD40‐AF700 signal. Data are expressed as mean ± standard deviation. Significance was analyzed by 2‐way ANOVA, ***p < 0.0005; ****p < 0.0001. Sidak correction was applied for multiple comparison. F) KPC tumor subcutaneous inoculation on right flank and treatment schematic. G) In vivo measurement of KPC tumor growth curve (*n* = 8/group). H) Bright field pictures of ex vivo tumors on day 14. I) Ex vivo tumor weight of all groups on day 14. J) Percentage change in tumor volumes on day 14 relative to their respective baselines (day 0). Each bar represents one mouse tumor. Data are expressed as mean ± standard deviation. Significance was analyzed by 2‐way ANOVA, ****p < 0.0001. The detailed significance between groups provided in Table S3, Supporting Information.

In this study, we used the NDES as a single‐administration extended‐released platform for intratumoral delivery of CD40 mAb in a slow and sustained manner. The NDES is an intratumoral implant smaller than a grain of rice, consisting of a stainless‐steel drug reservoir with a reservoir volume of 2 µL (**Figure** **1**A). Fundamental to the sustained drug release mechanism is a silicon nanofluidic membrane, which is mounted on one end of the implant (Figure 1A,B). The membrane presents seven circular microchannels. Each microchannel lead to 1400 nanochannels orthogonally etched through to the membrane surface (Figure 1A,B). The membrane is biocompatible and bioinert in vivo.^[^ ^30^ ^]^ Upon lyophilized drug loading into the reservoir, the other end of the implant is sealed using biocompatible silicone glue and primed in sterile phosphate‐buffered saline (PBS) for release. Drug release through the membrane is driven by concentration‐driven diffusive transport from the reservoir into the external environment. The release rate is controlled by confinement within the nanochannels, whereby their size and number can be altered for dose tuning.^[^ ^23^, ^31^ ^]^ In essence, drug release occurs through passive diffusion across the membrane, hence obviating the need for pumps or mechanics.^[^ ^23^, ^32^ ^]^ Release analysis showed a cumulative dose of 106 µg ± 20 µg of CD40 mAb over 14 days, indicating a daily release rate of 7.6 µg ± 1.4 µg (Figure 1C).

To demonstrate sustained release in vivo, NDES loaded with AlexaFluor 700 labeled CD40 (CD40‐AF700) were intratumorally implanted in KPC‐tumor bearing mice. AF700 fluorescent labeling allows for visualization of drug via IVIS imaging. As a control, a cohort of mice received a single‐dose intraperitoneal (IP) injection (100 µg of solubilized CD40‐AF700 in PBS). On days 1, 3, 7, and 14 post‐drug administration, tumors were harvested, and the presence of drug was assessed via total radiance efficiency measurements using ex vivo IVIS imaging analysis (Figure 1D,E). IVIS imaging analysis substantiated sustained intratumoral release from the NDES over 14 days, whereas drug penetration was apparent for IP cohort on day 1 and rapidly tapered off thereafter.

To assess treatment efficacy, KPC tumor‐bearing mice were randomized into two treatment arms: intraperitoneal (IP) and NDES (Figure 1F) to represent systemic and intratumoral delivery, respectively. On day 0, the IP cohort received either PBS as vehicle controls or 100 µg of solubilized CD40 mAb in PBS every other day for a total of 4 doses. Mice in the NDES arm were intratumorally implanted with devices preloaded with either PBS or ≈600 µg CD40 mAb via a one‐time minimally invasive trocar procedure on day 0. Upon intratumoral implantation, CD40 mAb or PBS autonomously diffuses out of the reservoir and into the tumor through the nanofluidic membrane in a controlled and sustained manner.

NDES CD40 treated mice presented significant tumor reduction comparable to that of IP CD40 cohort (Figure 1G). It is important to note that although similar tumor reduction was achieved, IP dosing was fourfold higher than NDES. The weight of explanted gross tumors at study endpoint on day 14 corroborated treatment efficacy of both NDES and IP‐delivered CD40 mAb (Figure 1H,I). Further, when assessing the percentage change in volume of each tumor at study endpoint (day 14) relative to its own baseline (day 0), we noted clear tumor regression in both treatment modalities, in comparison to control cohorts (Figure 1J).

In a separate study, CD40 mAb treatment using either systemic or intratumoral modalities improved the overall survival rate of KPC mice compared to their respective controls (Figure S2A, Supporting Information). Heterogenous treatment responses were evident between groups. However, complete response and tumor clearance were observed in some mice in both IP (*n* = 4/10 animals) and NDES (*n* = 2/10 animals) groups over the course of 90 days (Figure S2B–F, Supporting Information).

### 2.2 CD40 Monoclonal Antibodies Treatment Increased Immune Cells Infiltration and Reduced Fibrosis

**Figure 2:**
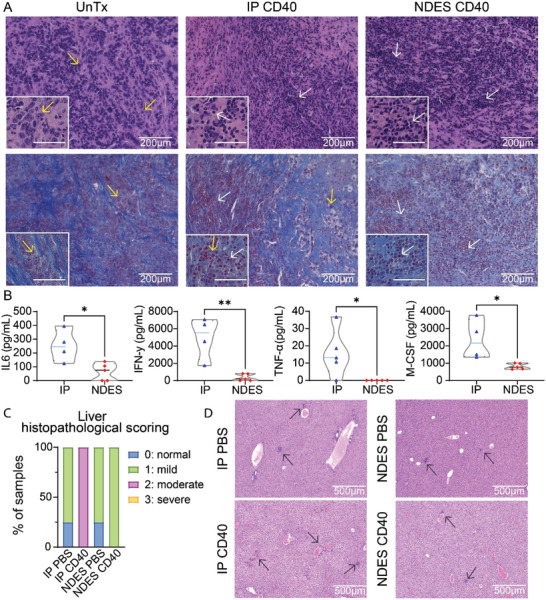
Histological analysis of tumors and treatment‐associated adverse events. A) H&E (top panel) and Masson's trichrome (bottom panel) staining shows the tumor composition of cancer cells (yellow arrows), fibroblast (blue staining), and infiltrating immune cells (white arrows). The length of scale bar for the bottom left image is 50 µm. B) Serum cytokine levels on day 1 as an indication of cytokine storm. *n* = 4/group. Data are expressed as mean ± standard deviation. Significance was analyzed by unpaired t‐test, *p < 0.05, **p < 0.005. C) Liver histopathology scoring. D) Representative H&E staining of liver samples. Black arrows indicate the inflamed loci.

PDAC tumors have characteristically dense fibrotic stroma, otherwise known as desmoplasia, which restricts immune cell and drug penetration.^[^ ^33^ ^]^ To visualize cellular infiltration (purple) and fibrosis (blue), respectively, tumor sections were stained with hematoxylin and eosin (H&E) and Masson's Trichrome (**Figure** **2**A). Untreated (UnTx) tumors prominently featured dense fibroblasts (bright blue) and cancer cells (light purple with enlarged nucleoli, representative yellow arrows). Regardless of administration modality, CD40 mAb treatment induced intratumoral immune cell infiltration (representative white arrows). Further, treated groups exhibited loose scar tissue (light blue) within tumor compared to denser fibrosis in UnTx samples.

To assess TRAE, we monitored the overall changes in mice body weight and rectal core temperature. Although body weight mildly reduced initially with IP CD40 treatment, mice recovered by day 4 (Figure S3A, Supporting Information). We observed no difference in the core temperature over the study duration (Figure S3B, Supporting Information).

Cytokine release syndrome (CRS), commonly known as cytokine storm is a serious immunological condition that can develop as an immunotherapy TRAE. In the clinic, cytokine storm can be life‐threatening and requires immediate care.^[^ ^34^ ^]^ We assessed the serum level of cytokine storm markers, interleukin 6 (IL6), interferon gamma (IFN‐*γ*), macrophage colony‐stimulating factor (M‐CSF), TNF‐*α*, one day after treatment with either IP or NDES CD40. Significantly high level of the cytokines was observed in the IP group compared to NDES cohort (Figure 2B), consistent with the adverse effects of systemic drug exposure.

CD40 agonist mAb is known to induce hepatotoxicity in the clinic.^[^ ^35^ ^]^ Organ‐specific toxicity was assessed as a function of liver inflammation via histologic scoring by a blinded board‐certified pathologist. Mice receiving IP CD40 presented moderate inflammation indicative of TRAE, whereas NDES group showed mild scoring similar to that of PBS controls (Figure 2C,D). To further investigate off‐tumor effects, we performed a systemic drug biodistribution analysis using IVIS, comparing a single‐dose IP injection to intratumoral NDES‐delivered CD40‐AF700. Off‐target drug exposure was detected in distant organs, particularly the liver, in the IP cohort at early time points (Figure S3C, Supporting Information). By day 14, total radiance values were similar to that of control organs, indicating drug clearance. These results were similar to our previous CD40 mAb biodistribution study in 4T1 tumor model.^[^ ^23b^ ^]^ Overall, these data suggest that the local controlled release of CD40 agonist mAb via NDES protected the mice from undesirable systemic drug exposure and its associated TRAE, presenting a safer treatment modality.

### 2.3 CD40 Monoclonal Antibodies Activated Local and Systemic Myeloid Cells Response

**Figure 3:**
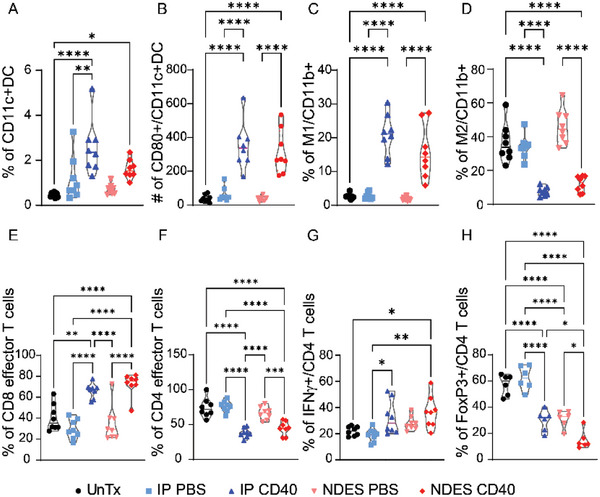
Tumor‐infiltrating immune cells assessment, including myeloid cells and lymphocytes on day 14. Myeloid cells population: A) DC, B) activated CD80+ DC, C) M1, and D) M2 cells. Lymphocytes population: E) CD8 effector T cells, F) CD4 effector T cells, G) IFN*γ*+ expressing CD4 T cells, and H) FoxP3+ Tregs via flow cytometry analysis. Data are expressed as mean ± standard deviation. Significance was analyzed by one‐way ANOVA. *p < 0.05; **p < 0.005; ***p < 0.0005; ****p < 0.0001. Tukey's correction was applied for multiple comparison.

CD40 is expressed on APCs including dendritic cells, macrophages, and B cells. Cancer antigens and differentiated functional immune cells can migrate to the tumor‐draining lymph node (TdLN) via circulatory or lymphatic systems to generate an abscopal anti‐tumor effects.^[^ ^36^ ^]^ As such, we assessed myeloid cellular responses systemically in the peripheral blood mononuclear cells (PBMC) and TdLN as well as locally within tumor. IP CD40 mAb treated cohort displayed increased DCs (CD11c+CD45+) in PBMC on days 3 and 7 post treatment; increase in NDES group was only observed on day 3 (Figure S4A, Supporting Information). Conversely, within the TIME, delivery of CD40 mAb via either modality achieved significantly higher DC infiltration (**Figure** **3**A) as well as activated DC (CD80+CD11c+CD45+) (Figure 3B) on day 14.

CD40 agonist mAb administration activates TAMs.^[^ ^37^ ^]^ Our study demonstrates that CD40 delivery via either IP or NDES significantly skews macrophages toward a M1 phenotypic response within the tumor after 14 days (Figure 3C,D). Additionally, although no difference in M1 population was observed across the groups in the TdLN, there was a statistically significant depletion of M2 cells on days 3 and 7 in NDES CD40 group (Figure S4B,C, Supporting Information). This indicates that intratumoral CD40 delivery can polarize macrophages locally toward a pro‐inflammatory M1 phenotype, possibly aiding with suppression of tumor growth.

Neutrophils have functional plasticity with either pro‐ or anti‐tumor affinity within the TIME.^[^ ^38^ ^]^ In murine models, neutrophil polarization can contribute to either accelerating or halting tumor progression.^[^ ^39^ ^]^ We demonstrate that CD40 treatment significantly increased neutrophil (Ly6G+Ly6C‐CD11b+) infiltration within tumor by on days 7 and 14 (Figure S5A, Supporting Information). Based on our tumor growth suppression data (Figure 1G), we hypothesize that the locally infiltrating neutrophils in this scenario could be tumor inhibitory. We further noted that IP delivery caused increase in systemic neutrophil infiltration in PBMC by day 14 (Figure S5B, Supporting Information). A similar effect was observed in TdLN of IP treated cohort on day 7 and normalized to that of UnTx by day 14 (Figure S5C, Supporting Information). We postulate that this robust neutrophil reaction could be indicative of an acute inflammatory reaction occurring in response to systemic CD40 mAb administration, as there were no substantial changes in the NDES cohort.

### 2.4 CD40 Monoclonal Antibodies Promotes Local Anti‐Tumor Immune Landscape

We observed CD40 administration significantly increased CD8+ effector T cell population (CD62L‐CD44+/CD8+) (Figure 3E). This effect was coupled with subsequent decrease of CD4+ effector T cells (CD62L‐CD44+/CD4+) compared to UnTx and respective control groups (Figure 3F). Further, CD40 administration increased IFN*γ* producing T cell subpopulation (Figure 3G), which could contribute to macrophage activation and enhanced anti‐tumor effects of CD8+ T cells.

We further examined the effect of CD40 and delivery modality on generation of immunosuppressive Tregs. Tregs are specialized CD4+ T cells (FoxP3+CD4+) that control immune tolerance and limit immune activation. The result indicated CD40 mAb administration decreased Treg population within the tumor compared to UnTx and their respective controls (Figure 3H). We noted that NDES control significantly decreased Treg phenotypes comparable to that of IP CD40.

### 2.5 CD40 Immunotherapy Enhanced TIL Infiltration and Converted Immunosuppressive Microenvironment

**Figure 4:**
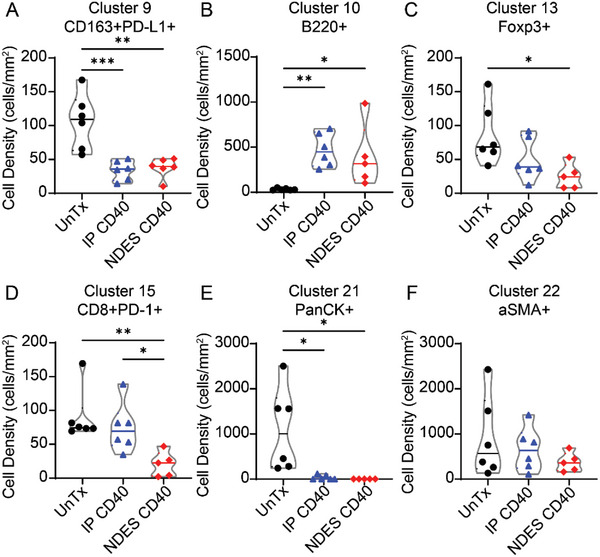
IMC analysis of TIME. Representative clusters from PhenoGraph. A) CD163+PD‐L1+ (M2 macrophages); B) B220+ (B cells); C) Foxp3+ (immunosuppressive intracellular marker); D) CD8+PD‐1+ (CD8+ exhausted T cells); E) PanCK+ (cancer cells); F) *α*SMA+ (endothelial cells). Cell density was averaged across 3 random fields of view per tumor (*n* = 2/group). Significance was analyzed by one‐way ANOVA. *p < 0.05; **p < 0.005; ***p < 0.0005. Tukey's correction was applied for multiple comparison.

Imaging mass cytometry (IMC) was used for a parallel analysis of paired image and cytometry analysis.^[^ ^40^ ^]^ Tumor sections from UnTx, IP CD40, and NDES CD40 groups were stained for immune cell spatial distribution. Twenty‐two phenotypes based on marker expression were clustered via PhenoGraph (Figures S6 and S7, Supporting Information) and exhibited on tSNE plots (Figure S7, Supporting Information). These clusters indicated TIME alteration due to the CD40 mAb treatment. Notably, CD40 mAb‐treated tumors showed a significant decrease of immunosuppressive M2 cell population (CD163+PD‐L1+) in the TIME (**Figure** **4**A). An increase of B cells infiltrated (B220+) in the TIME, which correlated to CD40 mAb treatment (Figure 4B), was noted. Tumor‐infiltrated B cells can potentially maintain beneficial anti‐tumor activity and shape the TIME for an adaptive immune response. On this note, a recent paper highlighted that antitumor efficacy of intratumoral immunotherapy is dependent on both T and B cells.^[^ ^41^ ^]^ Negatively regulated lymphocytes, detected using Foxp3+ or CD8+PD‐1+, showed decrease density in the CD40 mAb treated groups (Figure 4C,D). Moreover, the treated tumors showed decrease of cancer cells (PanCK+) and angiogenesis (*α*SMA+) in the TIME (Figure 4E,F). Overall, IMC analysis of the TIME suggested that CD40 mAb treatment transformed immunosuppressive TIME to immunogenic TIME.

**Figure 5:**
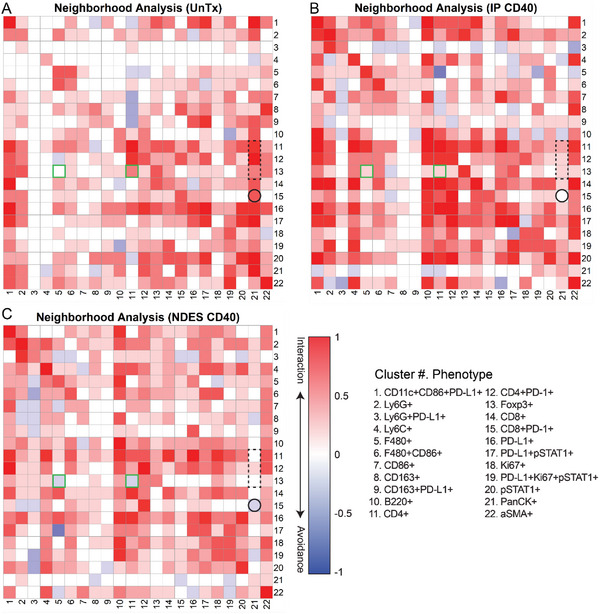
Neighborhood analysis of cellular interactions from IMC are presented as a heatmap. The color panel indicates significantly neighbored (red) or avoided (blue) cell‐cell interactions in A) UnTx, B) IP CD40, and C) NDES CD40 groups. Highlighted squares indicate directional interaction between cell phenotypes. Phenotypes #5 and #11 showed avoidance with phenotype #13 (green boxes) in NDES group compared to UnTx and IP CD40 groups. Phenotype #15 (CD8+PD1+) showed enrichment in cancer cells (#21, PanCK+) in UnTx TIME compared to NDES CD40 and IP CD40 groups (black circle). Phenotypes #11, 12, 13 (CD4+, CD4+PD1+, Foxp3+, respectively) showed significant enrichment in cancer cells (#21), in the UnTx group (dotted rectangle).

To further leverage the strength of spatial distribution analysis by IMC, we performed neighborhood analysis to determine the cell‐cell interaction (**Figure** **5**). The neighboring interaction between each pair of phenotypes is visualized as heatmap, in which each row and column indicates the neighborhood of a cell type of interest and the color represents the interaction or avoidance of a cell in the neighborhood. The immunosuppressive phenotype CD8+PD‐1+ was avoided in the cancer cells (PanCK+) neighborhood (black circle; row 15, column 21) in NDES CD40 and IP CD40 compared to UnTx. Moreover, a substantial enrichment of CD4+, CD4+PD‐1+, and Foxp3+ phenotypes cell populations near cancer cells were observed in UnTx compared to IP CD40 and NDES CD40 (dotted black rectangle; rows 11, 12, and 13, respectively, and column 21). The Foxp3+ phenotype cell population showed significant avoidance toward F480+ and CD4+ cell populations in NDES CD40 compared to IP CD40 and UnTx, which supports the therapeutic impact of local treatment on avoiding immunosuppressive TIME.

### 2.6 Addition of Radiotherapy to CD40 Immunotherapy Showed no Difference in Tumor Inhibition

Radiotherapy is a commonly used adjunctly to enhance oncotherapeutic effects. By triggering DNA damage, radiation induces damage‐associated molecular patterns to initiate APCs to license antigens, leading to priming of T cells and downstream cancer‐specific cytolytic immune responses.^[^ ^42^ ^]^ However, radiation may also functionally impair DCs^58^. In this scenario, impaired immunity can induce tolerance rather than activation.

**Figure 6:**
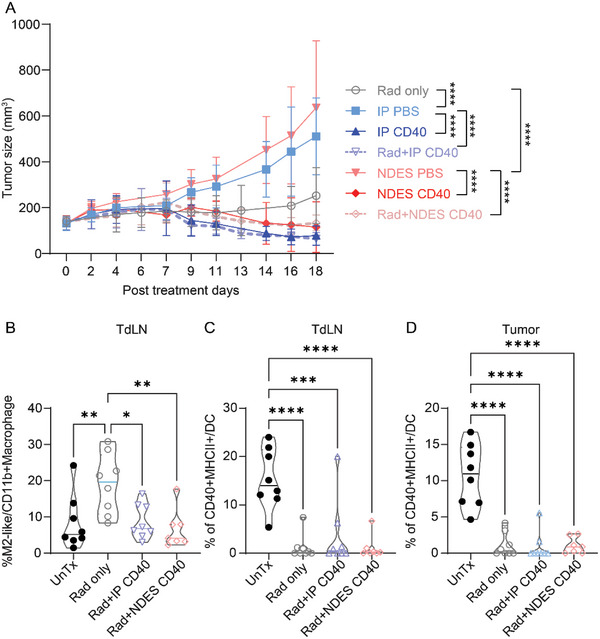
Combinational treatment efficacy of single dose of radiation and CD40 mAb treatment in KPC mice. A) Tumor growth curve (*n* = 7–8/group). Data are expressed as mean ± standard deviation. Significance was analyzed by two‐way ANOVA, ****p < 0.0001. The detailed significance between groups on each day was provided in Table S4, Supporting Information. Immune cells population in combination treated KPC mice: B) M2 macrophages in TdLN, and activated DCs in C) TdLN and D) tumor. Data are expressed as mean ± standard deviation. Significance was analyzed by one‐way ANOVA. *p < 0.05; **p < 0.005; ***p < 0.0005; ****p < 0.0001. Tukey's correction was applied for multiple comparisons.

To examine the synergistic effect of radiation with the NDES in PDAC, we administered a single dose of 10Gy^[^ ^26^ ^]^ radiation locally, one day prior to CD40 mAb treatment either via IP or NDES in KPC tumor‐bearing mice. Radiation was performed to induce immunogenic cell death to synergize with immunotherapy. Due to tumor heterogeneity and radio‐sensitivity between models, different radiation doses were used for each model. Notably, radiation treatment only slowed tumor growth rate at the beginning up to day 11 (**Figure** **6**A). Further, the addition of radiation to CD40 mAb did not significantly improve the rate of tumor burden reduction. Thus, we showed that radiotherapy did not augment immunotherapy in this model as previously hypothesized.

In mice receiving radiation only treatment, we observed increased the pro‐tumor M2‐like macrophages in TdLN compared to UnTx, whereas the addition of CD40 mAb treatment reduced this population (Figure 6B). Further, radiotherapy reduced the population of activated DCs in the TdLN and tumor (Figure 6C,D). Therefore, we postulate that radiation may direct the immune response toward immune tolerance and limit overall anti‐tumor activity in this murine model.

### 2.7 Local Treatment of CD40 Monoclonal Antibodies Demonstrated Abscopal Effect and Eliminated Distant Tumor

**Figure 7:**
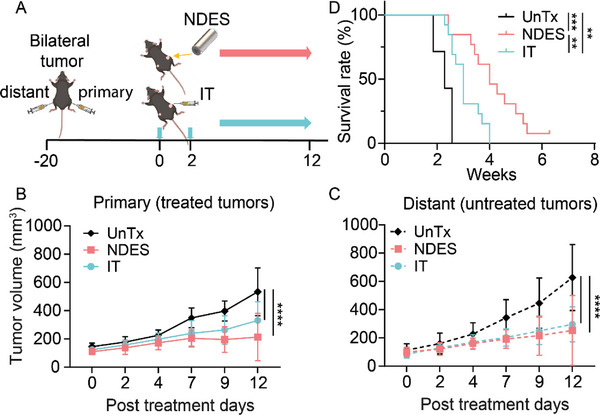
Bilateral KPC tumor model for assessing abscopal response. A) KPC tumor inoculation and treatment schematic on C57BL/6 mice. B) Growth curve of primary (treated) tumors. C) Growth curve of distant (UnTx) tumors. Data are expressed as mean ± standard deviation. Significance was analyzed by 2‐way ANOVA. ****p < 0.0001. The detailed significance between groups on each day is provided in Table S5, Supporting Information. Tukey's correction was applied for multiple comparison. D) Kaplan–Meier survival rate of UnTx, NDES, and IT groups. Significance was analyzed by log‐rank test; *n* = 8/group; **p < 0.001; ***p < 0.0005).

To evaluate whether local NDES CD40 mAb delivery could induce an abscopal effect, we used a bilateral KPC tumor model. In this study, we used intratumoral injection (IT) as a local treatment reference (**Figure** **7**A). IT group received injection of 10 µg CD40 mAb, each on days 0 and 2. Both primary and distant tumors responded to local treatment of either NDES or IT injection (Figure 7B,C). It is important to note that treatment response of the distant tumors was robust and comparable to that of the primary. However, both primary and distant tumors stopped responding to treatment after day 12.

Notably, NDES group demonstrated the potential of achieving an abscopal effect with complete tumor clearance observed in 1 out of 8 mice. In this mouse, both primary and distant tumors were eliminated, and it remained tumor‐free throughout the 100‐days of continued observation. Although abscopal effects were not achieved in other mice within the NDES group, Kaplan–Meier analysis showed that NDES group had significantly improved overall survival rates than those in the IT cohorts (Figure 7D). Moreover, we did not observe an abscopal effect in the IT group.

### 2.8 CD40 Monoclonal Antibodies Treatment Demonstrated Similar Response in Panc02 Murine Pancreatic Ductal Adenocarcinoma Model

**Figure 8:**
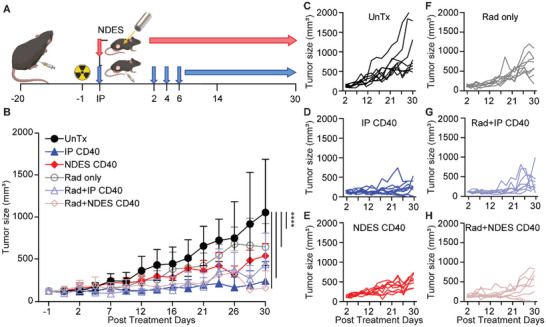
Panc02 tumor model for assessing CD40 mAb treatment administered via IP or NDES. A) Panc02 tumor inoculation and treatment schematic of C57BL/6 mice. Radiated groups received single 5 Gy dose one day prior to CD40 mAb treatment. B) In vivo tumor growth curve of each group. Significance was analyzed by 2‐way ANOVA and compared to UnTx group. ****p < 0.0001. IP CD40 versus NDES CD40 and Rad only: ****p < 0.0001; NDES CD40 versus Rad + IP CD40: ****p < 0.0001; NDES CD40 versus Rad + NDES CD40: ***p < 0.0005; Rad only versus Rad + IP CD40: ****p < 0.0001; Rad only versus Rad + NDES CD40: **p < 0.001. The detailed significance between groups on each day is provided in Table S6, Supporting Information. Tukey's correction was applied for multiple comparisons. C–H) Individual tumor growths of each group (*N* = 8/group).

An additional PDAC model, Panc02, was used to validate the treatment efficacy of sustained intratumoral CD40 mAb. Panc02 tumor‐bearing mice were randomized into treatment groups, UnTx, IP CD40, NDES CD40, Rad only, Rad + IP CD40, and Rad + NDES CD40. The radiated group received one dose of 5 Gy local radiation one day prior to CD40 mAb administration (**Figure** **8**A). Similar to the KPC study, radiation was performed to induce immunogenic cell death and synergize with immunotherapy and not intended to be curative. In the systemic delivery group, IP CD40 alone showed a prominent tumor reduction compared to Rad + IP CD40 group (Figure 8B). We surmise that radiation therapy potentially supported immunosuppressive TIME by depleting activated DCs in this cohort. Conversely, NDES delivered CD40 mAb showed the addition of radiation therapy significantly enhanced the tumor reduction (Figure 8C–H). The result indicated local CD40 mAb delivery via NDES could synergize with radiotherapy to augment local immune response and improve the treatment efficacy. Remarkably, in this PDAC model, two mice in the Rad + NDES CD40 group (*n* = 2 out of 8) achieved tumor clearance. Overall, we attribute the differences in response between KPC and Panc02 to the inherent disparities in TIME between the two models.^[^ ^26^ ^]^ Despite differences in TIME, our data supports the potential of NDES as a therapeutic modality.

## 3 Discussion

The success of immunotherapy in solid tumors has yet to be translated to PDAC, attributable to its low‐immunologic TIME.^[^ ^5^ ^]^ Several approaches are investigated to improve PDAC outcomes, including but not limited to GVAX (a vaccine‐based immunotherapy to enhance immune recognition of non‐immunogenic tumors), T cell adoptive therapy and agonist mAb.^[^ ^43^ ^]^ However, the optimal treatment strategy for targeting PDAC warrants further investigation. In this study, we used an innovative delivery approach to sustainably administer low‐dose agonist CD40 mAb intratumorally, which resulted in tumor elimination without adverse effects common to conventional systemic administration.

The CD40 signaling pathway is an important trigger for numerous immunological actions, including monocyte maturation and M1 macrophages differentiation, DC activation for enhancing antigen cross‐presentation to T cells, and mediating antibody switching and somatic hypermutation in B cells.^[^ ^44^ ^]^ A recent study reported that CD40 mAb reprograms TAM to anti‐tumor phenotype and accumulates tumor‐infiltrated immune cells along with TCR‐engineered T cells.^[^ ^45^ ^]^ Therefore, tuning the application of CD40 mAb to obtain the optimal treatment efficacy is crucial. Using the NDES for sustained intratumoral delivery, we demonstrated the reduction of M2 macrophages and increase of M1 macrophages systemically and locally, which are important for effective CD40 mAb treatment (Figure 3 and Figure S4, Supporting Information). Radiation dampened the presence of DC in the TIME and TdLN and induce the presence of M2 systemically in the KPC model; these immunosuppressive landscapes may explain the lack of synergistic improvement of treatment outcome when used in combination with CD40 mAb (Figure 6). However, the contradicting results in the Panc02 model (Figure 8) underscore that the heterogeneity in TIME play a role in treatment response, emphasizing the importance of personalized treatment based on individual patient tumor phenotypes.

TRAE occur depending on the agents used, administered dose and delivery method, which is typically systemic administration. Systemic delivery via infusion through a port or intravenous is a routine clinical practice, however drug toxicity is a primary concern.^[^ ^46^ ^]^ Although the combination of fully humanized CD40 mAb CP‐870893 and gemcitabine demonstrated therapeutic efficacy in patients with metastatic PDAC in a clinical trial, mild‐to‐moderate CRS, albeit transient (<24 h), occurred.^[^ ^27^ ^]^ Further, in another trial, weekly selicrelumab infusion was well‐tolerated, however some patients displayed chronic B cell activation, and marked decrease of CD4+ and CD8+ T cells when receiving maximum tolerated doses.^[^ ^43c^ ^]^ In line with this, intratumoral drug delivery is an emerging alternative approach for immunotherapy delivery. For example, a preclinical bladder cancer study of intratumoral agonist CD40 mAb showed anti‐tumor efficacy with reduced toxicity compared to systemic administration, which yielded high liver accumulation.^[^ ^17^ ^]^ A phase I clinical study evaluated intratumoral delivery of humanized IgG1 agonist CD40, ADC‐1013 in various solid tumors. Therapeutic effect was more favorable when injected into the lymph node or subcutaneous metastasis than deep metastasis, that is, liver, of solid tumors including melanoma, renal carcinoma, and ovarian adenocarcinoma, etc. However, 15 out of 18 patients experienced TRAE; the most commonly reported of any severity were pyrexia (39%), nausea (28%), vomiting (28%), fatigue (22%), influenza‐like illness (22%), chills (17%), and malaise (17%).^[^ ^47^ ^]^ These TRAE could be due to rapid dissemination into the circulation upon bolus intratumoral injection, mimicking the effects of systemic delivery.^[^ ^16c^ ^]^

Here we demonstrated that our NDES improves intratumoral delivery through slow and sustained controlled diffusive release, enhancing the therapeutic index.^[^ ^16c^ ^]^ The NDES achieved a similar efficacy as repeated systemic injections, albeit without TRAE. Notably, the NDES has a low‐dose release (≈7.6 µg per day) and only requires a one‐time minimally invasive trocar insertion procedure similar to brachytherapy. In contrast, systemic administration required repeated injections and high drug dosing (100 µg). Further, repeated systemic treatment showed evidence of TRAE inclusive of CRS and liver toxicities (Figure 2) as well as off‐tumor accumulation in non‐target organs (Figure S3C, Supporting Information).

CD40 mAb is shown to induce anti‐tumor immune response and turn the immunosuppressive environment to a more immunogenic TIME.^[^ ^48^ ^]^ Our results are supportive of this, where we observed DC activation, enhanced CD8+ T cells priming and CD4+ Tregs inhibition (Figure 3). Notably, local NDES treatment yielded an abscopal effect in a bilateral tumor model (Figure 7). Abscopal effect was first identified in radiotherapy, when irradiation of one tumor lead to regression of lesions at a distant site.^[^ ^49^ ^]^ However, the abscopal effect induced by radiotherapy was rarely observed in the clinic, which suggests that radiation alone is insufficient to overcome the immunosuppressive TIME. As the abscopal response is an immune‐mediated mechanism, there is a growing consensus that combining radiotherapy and immunotherapy has a great opportunity to boost anti‐tumor efficacy. A number of preclinical and clinical studies have shown increased abscopal response rates when combining dual anti‐CTLA4 and anti‐PD‐L1 or immunoadjuvants such as granulocyte‐M‐CSF with various radiotherapy dose and treatment timelines in solid tumors.^[^ ^50^ ^]^ In our study in KPC mice, we showed that sustained intratumoral delivery of CD40 mAb via NDES can achieve an abscopal effect without radiation. Although this result indicated the NDES CD40 mAb treatment overcame the immunosuppressive environment, it remains to be investigated whether the abscopal response can be amplified through the addition of radiation.

Finally, although we postulated that local delivery via the NDES could enhance immune infiltration across the desmoplastic tumor barrier, this was not the case. We observed similar efficacy in immune infiltration between systemic or intratumoral delivery. We note that future studies could entail the combination of immunotherapy with therapeutic agents to counter desmoplasia to support increased intratumoral immune infiltration. One caveat of our study is that the subcutaneous model may not fully recapitulate the TIME and provide high translational result akin to orthotopic models.^[^ ^51^ ^]^ However, this should not detract from our conclusion that sustained intratumoral delivery of CD40 mAb is a viable approach to replace systemic administration for effective anti‐tumor activity and reduced toxicity in PDAC. Further, our approach highlights the potential for a more standardized method for intratumoral treatment, overcoming the clinical issues of variability in administration techniques inclusive of needle gauge size and insertion angle as well as injection rate and volume.^[^ ^20^ ^]^

## 4 Experimental Section

### Nanofluidic Drug‐Eluting Seed Fabrication

NDES devices were fabricated as previously described.^[^ ^23^, ^24^ ^]^ Briefly, drug reservoir measuring 3 mm in length and 1.1 mm in diameter were machine‐cut and sanded from 18G 316 stainless tubes. Nanofluidic silicon membranes were microfabricated using silicon on insulator wafer.^[^ ^25^ ^]^ Membrane containing 250 nm nanochannels were affixed onto one end of the drug reservoir using biocompatible thermal epoxy (Epo‐TEK, 354‐T). Drug reservoir was filled with lyophilized CD40 mAb and sealed using silicon adhesive (Nusil, MED3‐4213) overnight at 4 °C. A layer of ultraviolet epoxy (Epo‐TEK, 0G116‐31) was applied over the silicone cap to prevent leakage. The implant was primed for drug release using sterile PBS under vacuum conditions.^[^ ^23a^ ^]^

### Murine Pancreatic Ductal Adenocarcinoma Models

Seven‐week‐old female C57BL/6 wild‐type mice were purchased from Taconic Biosciences (Rensselaer, NY) and housed at the Houston Methodist Research Institute (HMRI) Comparative Medicine under conditions outlined in the National Institutes of Health guide for Care and Use of Laboratory Animals and monitored daily. All animal studies were conducted in accordance with the protocol approved by the Institutional Animal Care and Use Committee (IACUC). KPC murine PDAC cell line was gifted from Dr. Sankar Mitra (Houston Methodist Research Institute) and cultured according to the guidelines. Panc02 murine PDAC cell line was acquired from the Division of Cancer Treatment and Diagnosis Tumor Repository program of NCI. KPC and Panc02 cells were confirmed to be mycoplasma negative prior to in vivo experimentation. For inoculation, either 1 × 10^6^ cells of KPC cells or 1 × 10^5^ cells of Panc02 cells were resuspended in 100 µL of 3:1 mixture of PBS and Matrigel (Corning, CB40234), then subcutaneously injected at the right flank. Tumor volume and mice weight were monitored thrice weekly with a digital caliper and scale. Tumor volume was calculated according to the formula: (Length × Width^2^)/2. The length was defined as the longest dimension and the width was defined as the perpendicular to the lengthwise axis.

### In Vivo Treatment

Once tumor volumes approximated ≈140 mm^3^, mice were randomized to different treatment arms. CD40 (FGK4.5; BE0016) mAb was purchased from Bio X Cell. Stock solution of CD40 was concentrated and lyophilized as described in previous protocol.^[^ ^23a^ ^]^ IP treatment groups were administered with either PBS (control; 100 µL) or lyophilized CD40 (100 µg in 100 µL of PBS) on days 0, 2, 4, and 6. NDES was filled with either PBS (control) or ≈600 µg lyophilized CD40 mAb. NDES devices were intratumorally implanted on day 0 using a minimally invasive trocar approach; no further intervention was performed for the NDES group. Over the course of the 14‐day study, NDES cohort received a total of 106.4 µg of CD40 mAb, whereas systemic group received a total of 400 µg of CD40 mAb.

For mice receiving radiation, mice were anesthetized with inhaled isoflurane and IP dexmedetomidine (5 µg g^−1^ body weight) before RT treatment. Radiation doses for KPC and Panc02 models were each selected based on literature references.^[^ ^26^ ^]^ RT was administered using the RS 2000 small animal irradiator (160 kV, 25 mA, and mean beam as 2 Gy min^−1^ Rad Source; Brentwood, TN) at 5 (Panc02) or 10 Gy (KPC) for 1 dose before immunotherapy treatment. Due to tumor heterogeneity and radio‐sensitivity between models, different radiation doses were used for each model. A lead layer was used to shield the mice from irradiation, exposing only the tumor. After RT, atipamezole was administered to reverse dexmedetomidine anesthesia.

### Percentage Change in Tumor Volume Relative to Baseline

The percentage of tumor growth was calculated by the percentage change of tumor volume at endpoint (*V*_t_) versus baseline treatment starting day (*V*_0_):^[^ ^27^ ^]^

(1) $$\%\Delta V_{t}=100\%\times \left(V_{t}-V_{0}\right)/V_{0}$$

The corresponding values were visualized as a waterfall plot.^[^ ^27^ ^]^ Positive values indicate increase in tumor size from baseline, whereas negative values indicate tumor reduction after treatment.

### Ex Vivo Assessment of CD40 Monoclonal Antibodies Biodistribution

CD40 mAb was concentrated using centricon tubes with a molecular weight cutoff of 30 kDa (Millipore). Filtrants were incubated with AlexaFluor 700 (AF700) N‐Hydroxysuccinimide ester at 4 °C overnight for fluorescent labeling. AF700‐abeled CD40 mAb was lyophilized with trehalose dehydrate at 37% w/w. The lyophilized CD40‐AF700 mAb were used to prepare IP injections or filled in the NDES. KPC‐bearing mice were randomized when tumor volumes approximated 140mm^3^. Radiated groups received a single dose 10 Gy prior to CD40 mAb treatment. The IP injection group received single dose of 100 µg of CD40‐AF700 in 100 µL of PBS, NDES group received CD40‐AF700 loaded devices via a minimally invasive one‐time intratumoral trocar insertion procedure. At endpoints, mice were euthanized by CO_2_ asphyxiation. Organs were harvested for ex vivo imaging via IVIS imaging system for biodistribution analysis of CD40‐AF700.

### Liver and Tumor Histologic Analysis

Tumor and liver were fixed in 10% formalin, embedded in paraffin, and sectioned at 5 µm. Liver sections were stained with hematoxylin and eosin (H&E), and tumor sections were stained with H&E and Masson's Trichrome at the Research Pathology Core of HMRI. H&E sections were evaluated by a pathologist blinded to treatment groups. Livers were assigned pathologic numeric scores according to literature, briefly as follows: 0, normal liver, no lesions or hepatocellular damage noted; 1, rare portal and parenchymal infiltrates but no necrosis; 2, moderate parenchymal or portal infiltrates but no necrosis; 3, frequent and/or large portal or parenchymal infiltrates with occasional isolated islands of coagulative necrosis; and 4, extensive areas of inflammation with bridging coagulative necrosis.

### Flow Cytometric Analysis

Tumor, TdLN, PBMC and spleen were collected for flow cytometry analysis at the study endpoint. The process is as described in literature.^[^ ^28^ ^]^ Tumors were mechanically dissociated into about 1 mm sections and incubated in RPMI‐1640 with 1x collagenase/hyaluronidase (StemCell Technologies, # 0 7912) and 20U mL^−1^ of DNase I (Sigma‐Aldrich, 112 849 320 001) for 1 h at 37 °C on a rocker. Single‐cell suspension was obtained via mechanical filtration through 70 µm cell strainers (Corning, 08‐771‐23). Tumor‐infiltrating leukocytes were separated using Lymphoprep (StemCell Technologuies, NC0665098). Spleens were dissociated into single‐cell suspension by mechanically filtration through 70 µm cell strainers and red blood cells were lysed with ACK lysis buffer (Quality biological, 118‐156‐101). TdLN were digested with in RPMI‐1640 with 1x collagenase/hyaluronidase and mechanically filtered through 70 µm cell strainers. Cells were washed with PBS and resuspended in FACS buffer (PBS with 2% FBS) then plated in 96‐well U‐bottom plate. Cells were stained with CD16/CD32 (BD Bioscience, 553 141) for 30 min, and stained using either myeloid or T cell panel (Table S1, Supporting Information). Events were acquired on LSRII (BD Biosciences) flow cytometer and analyzed using FlowJo v10.7.2 software. Detailed gating strategies for myeloid and lymphoid cells are included in Figure S1, Supporting Information.

### Imaging Mass Cytometry and Neighborhood Analysis

Metal‐labeled antibodies were prepared according to the Fluidigm protocol for tumor section staining. Antibodies used in this study are listed in Table S2, Supporting Information. Details are as previously described.^[^ ^23a^ ^]^ The sections were ablated with Hyperion (Fluidigm) for data acquisition. IMC data were segmented by ilastik and CellProfiler. Histology topography cytometry analysis toolbox (HistoCAT) and R scripts were used to quantify cell number, generate tSNE plots, and perform neighborhood analysis.^[^ ^29^ ^]^ After permutation tests of 1000 runs, the neighborhood status of pairwise interactions between cell phenotypes in each sample are denoted interaction (1), avoidance (−1), or undetermined (0). Mann–Whitney *U* test were further applied to statistically test the denoted neighborhood status across groups (https://www.ncbi.nlm.nih.gov/books/NBK560699/), tumor and cellular densities were averaged across 3 images per tumor, with *n* = 2 per group.

### Bilateral Murine KPC Tumor Model

Syngeneic bilateral KPC tumors were inoculated subcutaneously on the right flank (1 × 10^6^ cells) and 3 days later, on the left flank (5 × 10^5^ cells) in female C57BL/6 wild‐type mice. Tumors were monitored and measured as described above. When tumor volumes on the right flank reached ≈140 mm^3^, mice were randomized into UnTx, IT, and NDES treatment groups (*n* = 8/group). UnTx group received no treatment, IT group received two injections of 10 µg CD40 mAb spaced three days apart, NDES group each had one device implantation with release rate of ≈7.6 µg CD40 mAb per day. Mice were euthanized when the sum of both tumor volumes exceeded humane endpoint.

### Cytokine Serum ELISA

The levels of IL‐6, TNF*α*, M‐CSF, and IFN*γ* in the serum were measured via ELISA one day post treatment. Blood was extracted via cardiac puncture and collected in BD Microtainer blood collection tubes (with serum separating gel). Serum was isolated after centrifugation at 2000 × *g* for 10 min. Mouse IL‐6 (# 431 304), TNF*α* (# 430 904), M‐CSF(# 448 504), and IFN*γ* (# 430 804) were measured following the manufacture protocol of ELISA MAX Deluxe Set from Biolegend.

### Statistical Analysis

All data were analyzed using GraphPad Prism 9.1.2. For parametric data sets, statistical significance was determined by unpaired t test for 2‐tailed data. Unpaired 1‐way, 2‐way analysis of variance (ANOVA) and multiple comparisons were performed using Tukey corrections. Kaplan–Meier method was used for survival analysis. The overall survival was calculated using the Log‐rank (Mantel‐Cox) test. Statistical significance was determined as *p < 0.05; **p < 0.005; ***p < 0.0005; ****p < 0.0001.

## Conflict of Interest

The authors declare no conflict of interest.

## Supporting information

Supporting Information

## Data Availability

The data that support the findings of this study are available from the corresponding author upon reasonable request.
