# Supplementary material for: Sustained Intratumoral Administration of Agonist CD40 Antibody Overcomes Immunosuppressive Tumor Microenvironment in Pancreatic Cancer
Source: Adv Sci (Weinh). 2023 Jan 19;10(9):2206873. doi: 10.1002/advs.202206873 (PMC10037694; doi:10.1002/advs.202206873)
Supplement: Supplementary file 1 — Supporting Information [file ADVS-10-2206873-s001.pdf]

ADVANCED SCIENCE
Open Access

Supporting Information

for *Adv. Sci.*, DOI 10.1002/advs.202206873

Sustained Intratumoral Administration of Agonist CD40 Antibody Overcomes Immunosuppressive Tumor Microenvironment in Pancreatic Cancer

Hsuan-Chen Liu, Daniel Davila Gonzalez, Dixita Ishani Viswanath, Robin Shae Vander Pol, Shani Zakiya Saunders, Nicola Di Trani, Yitian Xu, Junjun Zheng, Shu-Hsia Chen, Corrine Ying Xuan Chua and Alessandro Grattoni**

Supplementary Data

Sustained intratumoral administration of agonist CD40 antibody overcomes immunosuppressive tumor microenvironment in pancreatic cancer

Hsuan-Chen Liu[1], Daniel Davila Gonzalez[1], Dixita Ishani Viswanath[1,2], Robin Shae Vander Pol[1], Shani Zakiya Saunders[1], Nicola Di Trani[1], Yitian Xu[3,4], Junjun Zheng[3,4], Shu-Hsia Chen[3,4], Corrine Ying Xuan Chua[1*], and Alessandro Grattoni[1,5,6*]

Supplemental Table S1

List of antibodies for flow cytometry analysis

Antibody	fluorophore	clone	cat no.	brand
LIVE/DEAD™ Fixable Blue Dead Cell Stain	DAPI		L34962	ThermoFisher
CD45	APC-cy7	30-F11	103224	Biolegend
CD11b	BV650	M1/70	563015	BD Biosciences
CD11c	PerCP-Cy5.5	N418	117327	Biolegend
MHC-II (IA/IE	BV510	M5/114.1	107636	Biolegend
CD80	BV786	16-10A1	740888	BD Biosciences
Ly6G	BV711	1A8	127643	Biolegend
Ly6C	APC	HK1.4	17-5932-82	ThermoFisher
CD206	PE-Cy7	MR6F3	25-2061-82	eBioscience™
F4/80	PE-eFluor 610	BM8	61-4801-82	eBioscience™
CD3	APC-eFluor® 780	145-2C11	47-0031-82	eBioscience™
CD4	BV605	RM4-5	BDB563151	BD Biosciences
CD8	PerCP-Cy5.5	53-6.7	45-0081-82	eBioscience™
CD69	PE-Cy7	H1.2F3	25-0691-82	eBioscience™
CD44	PE	IM7	103007	Biolegend
CD62L	APC	MEL-14	104412	Biolegend
PD-1	BV711	J43	744547	BD Biosciences
CD25	PE-CF594	PC61	562694	BD Biosciences
ki67	eFluor 506	SolA15	69-5698-82	eBioscience™
IFN-r	APC	XMG1.2	505810	Biolegend
FOXP3	PE-cy7	FJK-16s	25-5773-82	eBioscience™
Granzyme B	PE	NGZB	12-8898-82	eBioscience™

Supplemental Table S2

IMC staining panel

Antibody Name	Label
CD8-alpha	Dy162
CD163	Sm152
CD4	Yb174
PD-1	Tm169
CD11b	Nd146
CD11c	Sm149
Ly6C	Nd144
Ly6G	Nd145
CD86	Lu175
PD-L1	Dy164
Foxp3	Gd160
F4/80	Yb176
PanCK	Nd148
a-SMA	Sm147
pSTAT1	Gd155
Ki-67	Er167
B220	Er170

Supplemental Table S3.
Statistical significance table for Fig 1G.

Day 9	Untreated	IP PBS	IP CD40	NDES PBS	NDES CD40
Untreated					
IP PBS	ns				
IP CD40	*	ns			
NDES PBS	ns	ns	**		
NDES CD40	**	ns	ns	***	

Day 11	Untreated	IP PBS	IP CD40	NDES PBS	NDES CD40
Untreated					
IP PBS	ns				
IP CD40	****	***			
NDES PBS	ns	ns	****		
NDES CD40	****	**	ns	****	

Day 14	Untreated	IP PBS	IP CD40	NDES PBS	NDES CD40
Untreated					
IP PBS	ns				
IP CD40	****	****			
NDES PBS	ns	ns	****		
NDES CD40	****	****	ns	****	

Supplemental Table S4.
Statistical significance table for Fig 6.

Day 9	IP PBS	IP CD40	NDES PBS	NDES CD40	Rad only	Rad IP CD40	Rad NDES CD40
IP PBS							
IP CD40	ns						
NDES PBS	ns	**					
NDES CD40	ns	ns	ns				
Rad only	ns	ns	ns	ns			
Rad IP CD40	*	ns	ns	ns	ns		
Rad NDES CD40	ns	ns	***	ns	ns	ns	

Day 9	IP PBS	IP CD40	NDES PBS	NDES CD40	Rad only	Rad IP CD40	Rad NDES CD40
IP PBS							
IP CD40	**						
NDES PBS	ns	***					
NDES CD40	ns	ns	*				
Rad only	ns	ns	**	ns			
Rad IP CD40	**	ns	****	ns	ns		
Rad NDES CD40	*	ns	**	ns	ns	ns	

Day 14	IP PBS	IP CD40	NDES PBS	NDES CD40	Rad only	Rad IP CD40	Rad NDES CD40
IP PBS							
IP CD40	****						
NDES PBS	ns	****					
NDES CD40	****	ns	****				
Rad only	***	ns	****	ns			
Rad IP CD40	****	ns	****	ns	ns		
Rad NDES CD40	****	ns	****	ns	ns	ns	

Day 16	IP PBS	IP CD40	NDES PBS	NDES CD40	Rad only	Rad IP CD40	Rad NDES CD40
IP PBS							
IP CD40	****						
NDES PBS	ns	****					
NDES CD40	****	ns	****				
Rad only	****	*	****	ns			
Rad IP CD40	****	ns	****	ns	*		
Rad NDES CD40	****	ns	****	ns	ns	ns	

Day 18	IP PBS	IP CD40	NDES PBS	NDES CD40	Rad only	Rad IP CD40	Rad NDES CD40
IP PBS							
IP CD40	****						
NDES PBS	ns	****					
NDES CD40	****	ns	****				
Rad only	****	***	****	*			
Rad IP CD40	****	ns	****	ns	***		
Rad NDES CD40	****	ns	****	ns	ns	ns	

Supplemental table S5.

Statistical significance table for Figs 7B.

Day 7	UnTx	NDES	IT
UnTx			
NDES	*		
IT	*	ns	

Day 9	UnTx	NDES	IT
UnTx			
NDES	****		
IT	***	ns	

Day 12	UnTx	NDES	IT
UnTx			
NDES	****		
IT	****	ns	

Statistical significance table for Figs 7C.

Day 7	UnTx	NDES	IT
UnTx			
NDES	*		
IT	*	ns	

Day 9	UnTx	NDES	IT
UnTx			
NDES	****		
IT	***	ns	

Day 12	UnTx	NDES	IT
UnTx			
NDES	****		
IT	****	ns	

Supplemental table S6.
Statistical significance table for Fig 8.

Day 12	UnTx	IP CD40	NDES CD40	Rad only	Rad IP CD40	Rad NDES CD40
UnTx						
IP CD40	ns					
NDES CD40	ns	ns				
Rad only	ns	ns	ns			
Rad IP CD40	*	ns	ns	ns		
Rad NDES CD40	ns	ns	ns	ns	ns	

Day 14	UnTx	IP CD40	NDES CD40	Rad only	Rad IP CD40	Rad NDES CD40
UnTx						
IP CD40	**					
NDES CD40	ns	ns				
Rad only	ns	ns	ns			
Rad IP CD40	*	ns	ns	ns		
Rad NDES CD40	*	ns	ns	ns	ns	

Day 16	UnTx	IP CD40	NDES CD40	Rad only	Rad IP CD40	Rad NDES CD40
UnTx						
IP CD40	*					
NDES CD40	ns	ns				
Rad only	ns	ns	ns			
Rad IP CD40	**	ns	ns	ns		

Rad NDES CD40	ns	ns	ns	ns	ns	

Day 19	UnTx	IP CD40	NDES CD40	Rad only	Rad IP CD40	Rad NDES CD40
UnTx						
IP CD40	***					
NDES CD40	ns	ns				
Rad only	ns	*	ns			
Rad IP CD40	**	ns	ns	ns		
Rad NDES CD40	**	ns	ns	ns	ns	

Day 21	UnTx	IP CD40	NDES CD40	Rad only	Rad IP CD40	Rad NDES CD40
UnTx						
IP CD40	****					
NDES CD40	**	ns				
Rad only	ns	*	ns			
Rad IP CD40	****	ns	ns	ns		
Rad NDES CD40	****	ns	ns	ns	ns	

Day 23	UnTx	IP CD40	NDES CD40	Rad only	Rad IP CD40	Rad NDES CD40
UnTx						
IP CD40	****					
NDES CD40	**	ns				
Rad only	ns	**	ns			
Rad IP CD40	***	ns	ns	ns		

Rad NDES CD40	****	ns	ns	ns	ns	

Day 26	UnTx	IP CD40	NDES CD40	Rad only	Rad IP CD40	Rad NDES CD40
UnTx						
IP CD40	****					
NDES CD40	****	ns				
Rad only	ns	****	***			
Rad IP CD40	***	ns	ns	**		
Rad NDES CD40	****	ns	ns	**	ns	

Day 28	UnTx	IP CD40	NDES CD40	Rad only	Rad IP CD40	Rad NDES CD40
UnTx						
IP CD40	****					
NDES CD40	****	*				
Rad only	*	****	ns			
Rad IP CD40	****	ns	ns	**		
Rad NDES CD40	****	ns	ns	**	ns	

Day 30	UnTx	IP CD40	NDES CD40	Rad only	Rad IP CD40	Rad NDES CD40
UnTx						
IP CD40	****					
NDES CD40	****	**				
Rad only	****	****	ns			

Rad IP CD40	****	ns	ns	*		
Rad NDES CD40	****	ns	ns	ns	ns	

Supplemental sFig 1.

Myeloid cells gating strategy

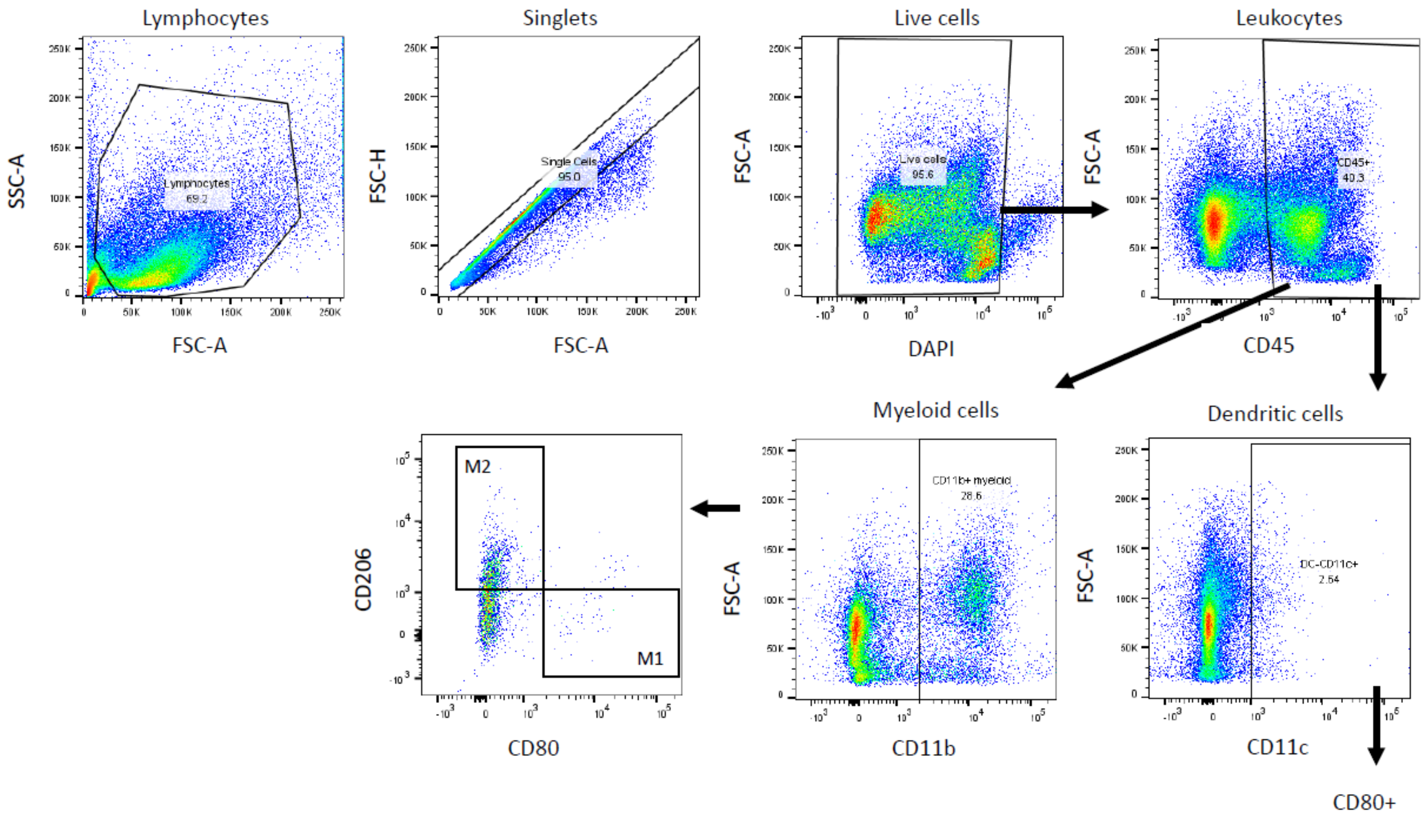

Lymphoid cells gating strategy

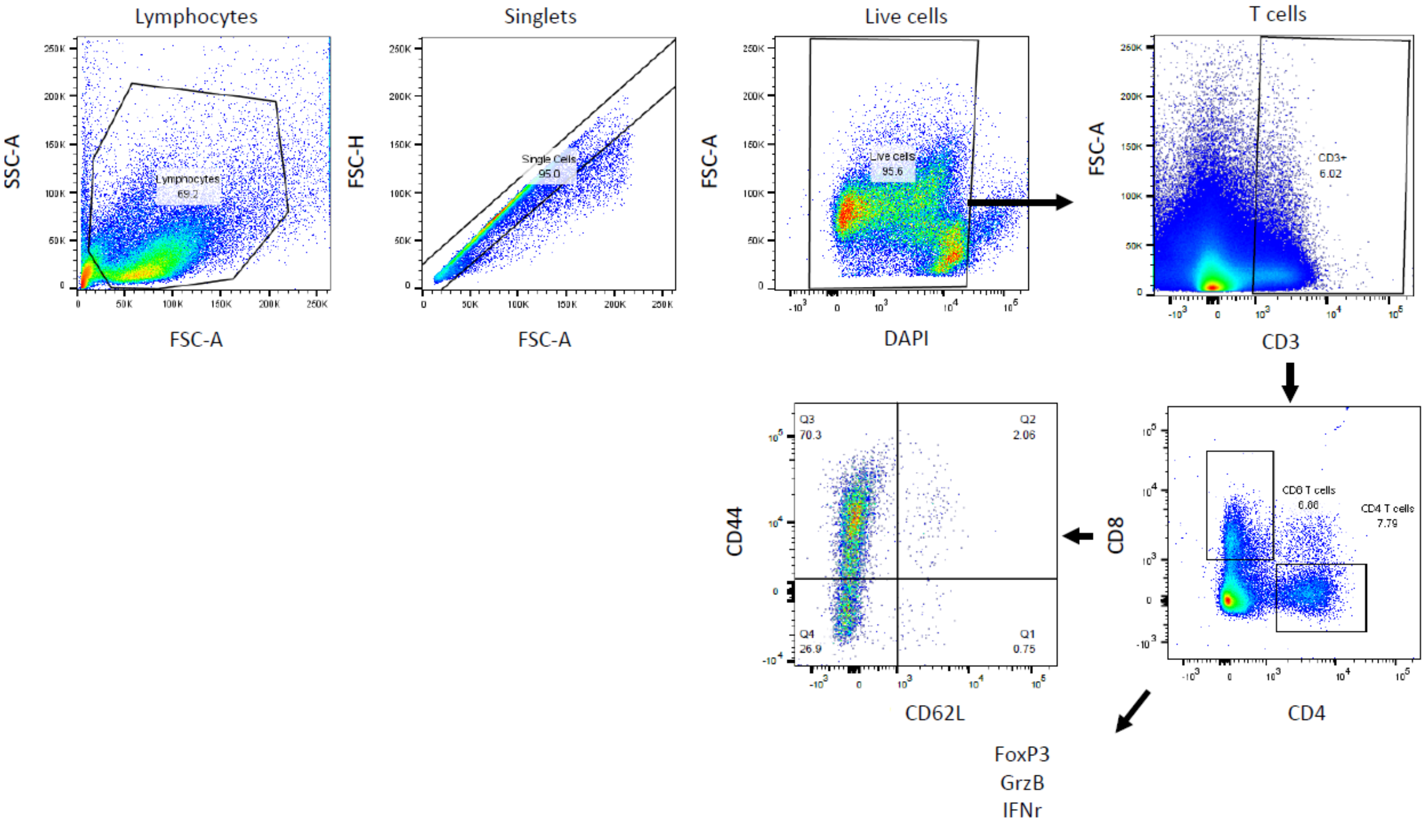

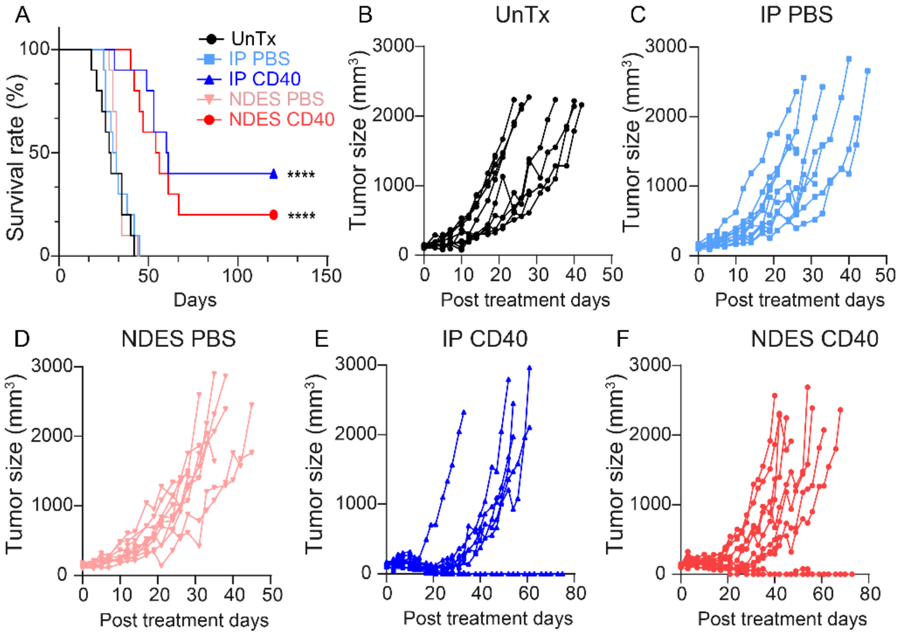

Supplemental SFig 2. A) Kaplan-Meier curves comparing overall survival rates of mice treated with IP or NDES-delivered CD40 (log-rank test; n=10/ group; **** P<0.0001). B-F) Individual tumor growth of each group.

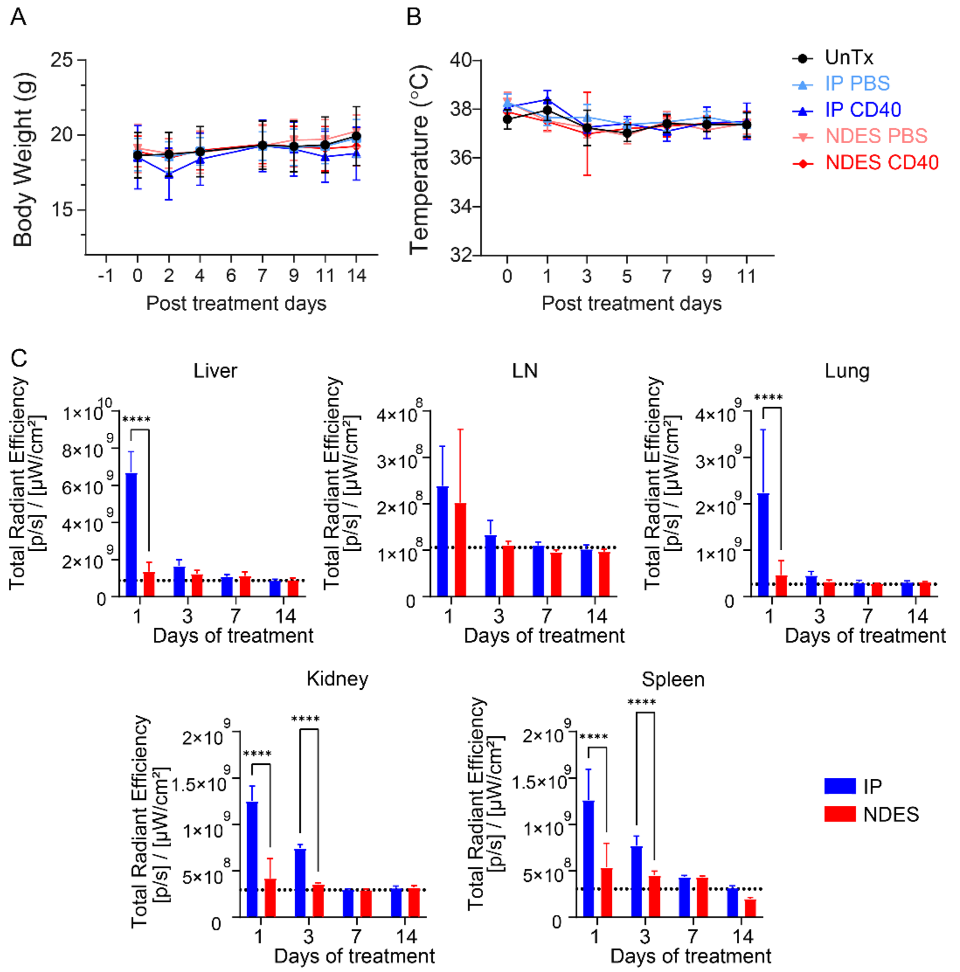

Supplemental SFig 3. Mice A) total body weight and B) rectal core temperature. N=8/group. C) Biodistribution of CD40-AF700 in the organs of KPC tumor-bearing mice that received a single administration of CD40-AF700, alone or in combination with a single dose radiation. Organs included kidneys, draining LN, lung, liver and spleen. The dash lines indicated the total radiance values of control organs that did not receive CD40-AF700. Data are expressed as mean ± standard deviation. Significance was analyzed by 2-way ANOVA, *** P<0.0005; **** P<0.0001. Sidak correction was applied for multiple comparison.

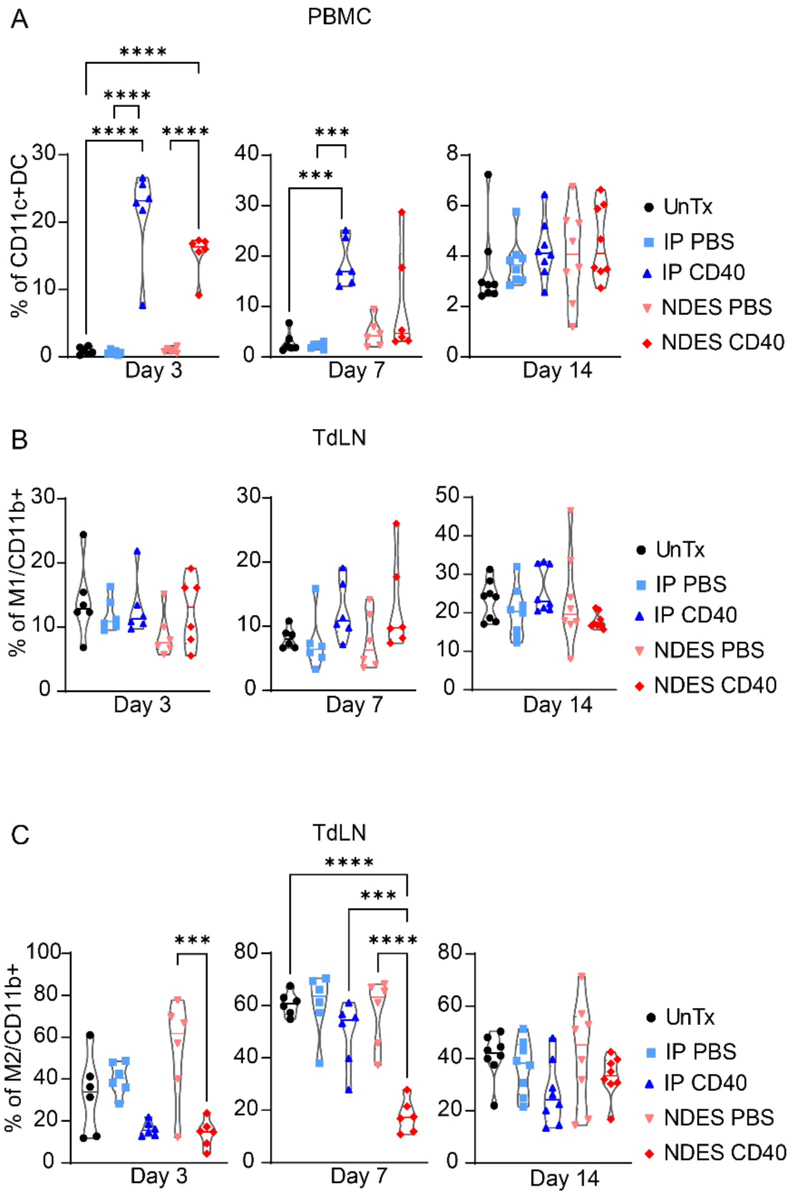

Supplemental SFig 4. Myeloid cells assessment systemically (PBMC and TdLN) and locally (tumor). A) DC cell population assessment in PBMC at multiple time points. B) M1 and C) M2 cell population in TdLN at multiple time points. Data are expressed as mean ± standard deviation. Significance was analyzed by one-way ANOVA. * P<0.05; ** P<0.005; *** P<0.0005; **** P<0.0001. Tukey's correction was applied for multiple comparison.

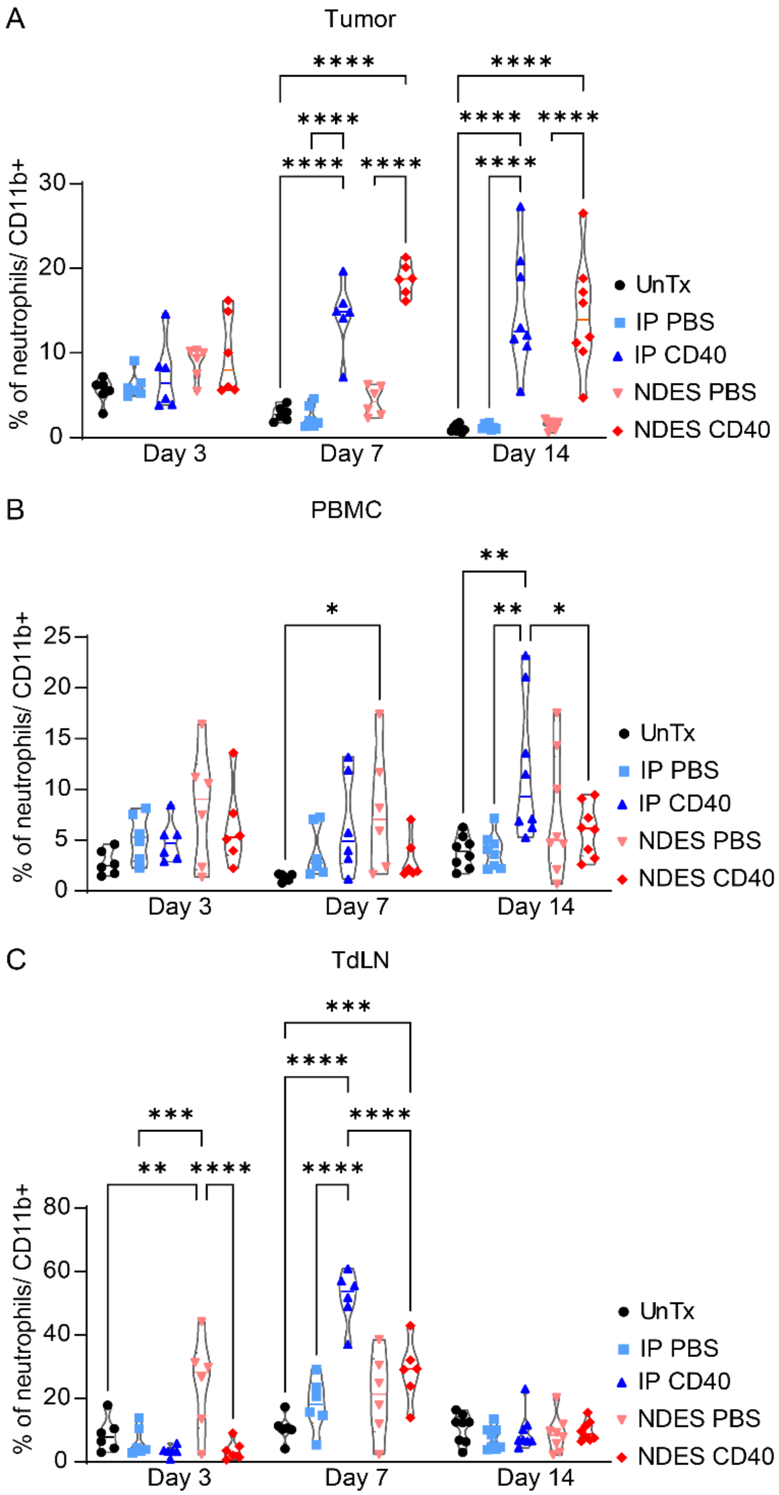

Supplemental SFig 5. Neutrophil populations in A) tumor, B) PBMC and C) TdLN across multiple time points. Data are expressed as mean ± standard deviation. Significance was analyzed by one-way ANOVA. * P<0.05; ** P<0.005; *** P<0.0005; **** P<0.0001. Tukey's correction was applied for multiple comparison.

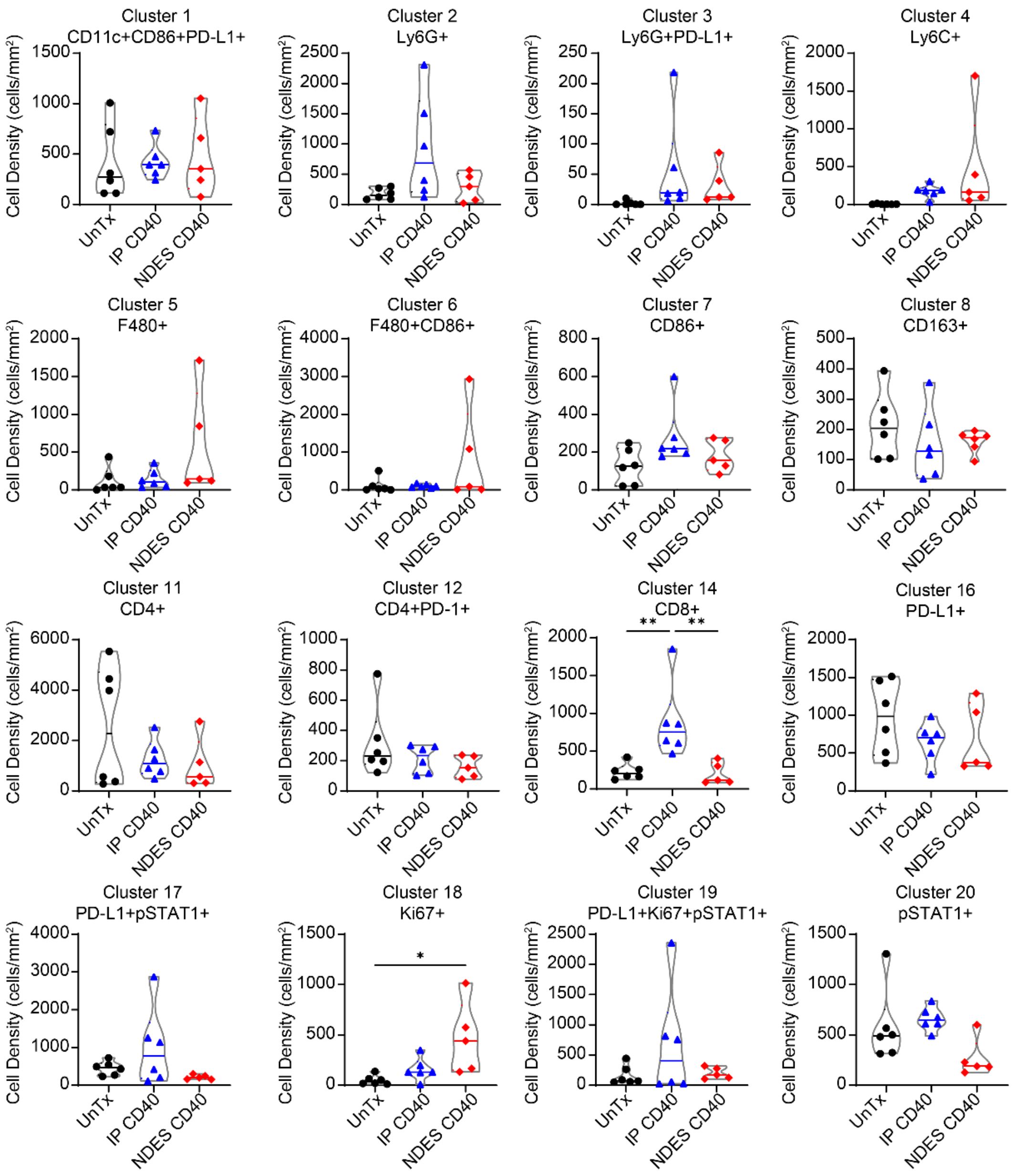

Supplemental SFig 6. IMC clustering via PhenoGraph. Significance was analyzed by one-way ANOVA. * P<0.05; ** P<0.005. Tukey's correction was applied for multiple comparison.

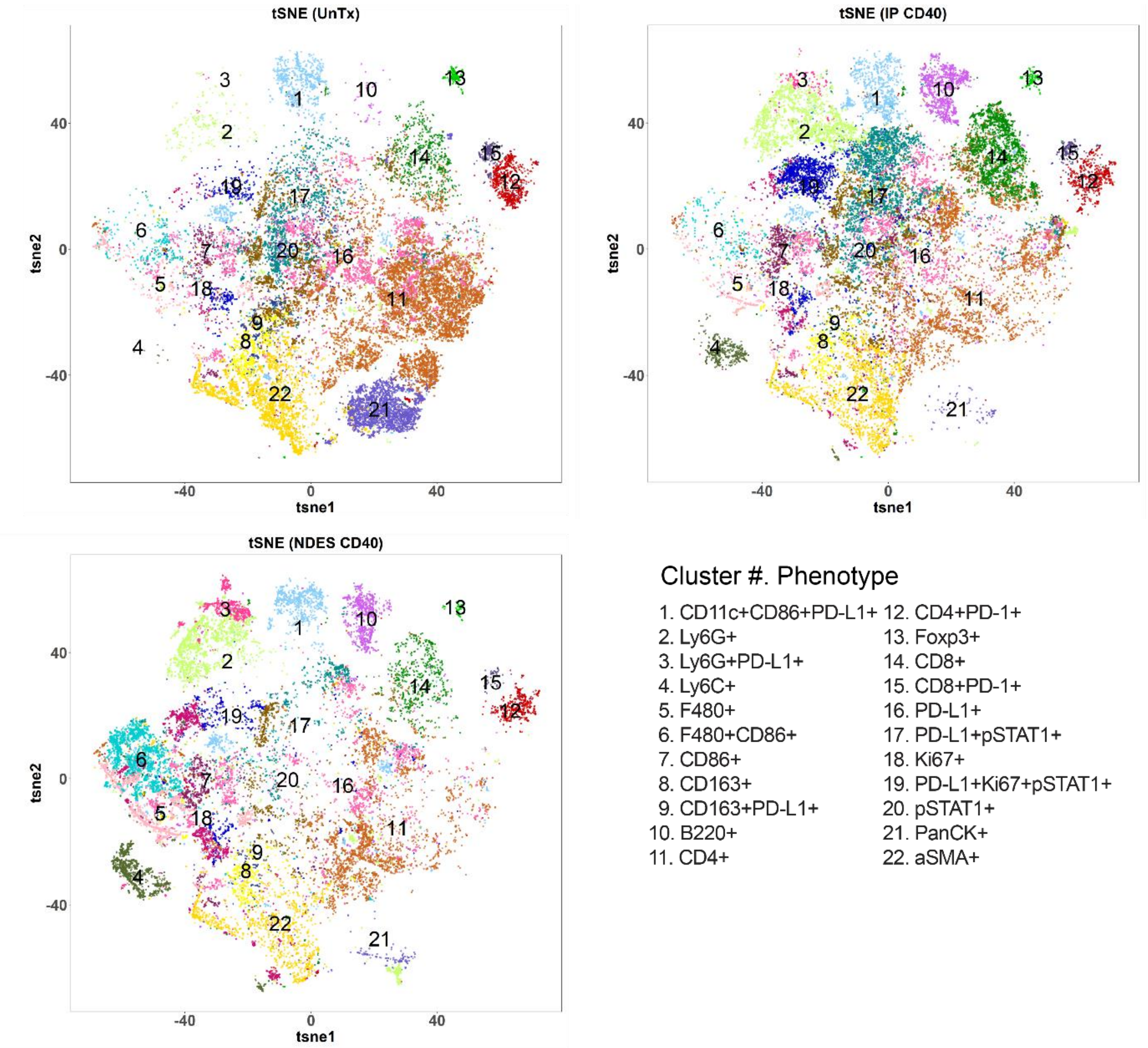

Supplemental SFig 7. The visualized multi-dimensional reduction tool-tSNE map of each group.
